# Effects of parietal TMS on somatosensory judgments challenge interhemispheric rivalry accounts

**DOI:** 10.1016/j.neuropsychologia.2010.07.031

**Published:** 2010-10

**Authors:** Neir Eshel, Christian C. Ruff, Bernhard Spitzer, Felix Blankenburg, Jon Driver

**Affiliations:** a UCL Institute of Cognitive Neuroscience, University College London, 17 Queen Square, London WC1N 3AR, UK; b Harvard Medical School, Boston, MA 02115, USA; c Wellcome Trust Centre for Neuroimaging at UCL, Institute of Neurology, University College London, 12 Queen Square, London WC1N 3BG, UK; d Laboratory for Social and Neural Systems Research, IEW, University of Zurich, Zurich, Switzerland; e Department of Neurology and Bernstein Center for Computational Neuroscience, Charité, Philippstr. 13, House 6, 101115 Berlin, Germany

**Keywords:** Transcranial magnetic stimulation, Rivalry, Tactile perception

## Abstract

Interplay between the cerebral hemispheres is vital for coordinating perception and behavior. One influential account holds that the hemispheres engage in rivalry, each inhibiting the other. In the somatosensory domain, a seminal paper claimed to demonstrate such interhemispheric rivalry, reporting improved tactile detection sensitivity on the right hand after transcranial magnetic stimulation (TMS) to the right parietal lobe (Seyal, Ro, & Rafal, 1995). Such improvement in tactile detection ipsilateral to TMS could follow from interhemispheric rivalry, if one assumes that TMS disrupted cortical processing under the coil and thereby released the other hemisphere from inhibition. Here we extended the study by Seyal et al. (1995) to determine the effects of right parietal TMS on tactile processing for either hand, rather than only the ipsilateral hand. We performed two experiments applying TMS in the context of median-nerve stimulation; one experiment required somatosensory detection, the second somatosensory intensity discrimination. We found different TMS effects on detection versus discrimination, but neither set of results followed the prediction from hemispheric rivalry that enhanced performance for one hand should invariably be associated with impaired performance for the other hand, and vice-versa. Our results argue against a strict rivalry interpretation, instead suggesting that parietal TMS can provide a pedestal-like increment in somatosensory response.

## Research highlights

▶ TMS to right parietal cortex improves ipsilateral somatosensory detection. ▶ The same TMS bursts impair bilateral somatosensory discrimination. ▶ The results argue against interhemispheric rivalry. ▶ A possible explanatory model includes excitatory interhemispheric interactions.

## 1 Introduction

The cerebral hemispheres are constantly interacting. More than 200 million axons transmit information between homologous regions on opposite sides, making the corpus callosum the brain's largest connective tract (Banich, 1995; Tomasch, 1954). Lesions to this tract—whether due to disease, surgery, or congenital condition—result in a complex set of deficits, indicating that interhemispheric connections are vital to coordinate sensory, cognitive, and motor processing (for review, see Gazzaniga, 2005).

Somatosensation is one domain that is particularly affected by interhemispheric exchanges. It has long been known that each half of the body-surface projects information initially to the contralateral hemisphere of the brain (Maldjian, Gottschalk, Patel, Detre, & Alsop, 1999; Penfield & Rasmussen, 1950). Neurons in primary sensory cortex do not receive input directly from ipsilateral stimuli. However, if information from a given body-part is blocked through an intervention such as local anesthesia, the brain appears to undergo a two-stage response (Calford & Tweedale, 1990). As expected, neurons that normally represent the denervated region expand their receptive fields to represent nearby regions. But at the same time, neurons *ipsilateral* to the anesthesia can expand their receptive fields as well, mirroring the contralateral expansion. Clearly, information has transferred between the hemispheres, perhaps to enable balanced processing.

The fundamental observation that somatosensory processing in one hemisphere can modulate somatosensory processing in the other hemisphere has now arisen in many contexts. These range from invasive studies in monkeys (e.g., Clarey, Tweedale, & Calford, 1996), to behavioral or neuroimaging studies of healthy humans (e.g., Hlushchuk & Hari, 2006) or patients with brain damage (e.g., Oliveri et al., 1999).

For more than 30 years, a dominant account of interhemispheric interactions has posited that the hemispheres engage in a ‘seesaw-type’ *rivalry* (Kinsbourne, 1977). According to this account, each hemisphere deals with and attends primarily to the opposite side of sensory space, while inhibiting the capacity of the other hemisphere to do likewise. If one hemisphere is damaged or disrupted, the intact side is thought to be released from inhibition, resulting not only in deficient attention for space contralateral to the lesion but also potentially in excessive attention ipsilesionally. In other words, the intact side comes to dominate the disrupted one.

Over the years this hemispheric *rivalry* account has received some support from both the human and animal literature. It may help to explain unilateral neglect, the clinical phenomenon in which patients with unilateral lesions (especially in and around the right posterior parietal cortex) ignore stimuli located opposite the lesion, despite intact primary sensory machinery (for review, see Driver & Mattingley, 1998). In particular, the hemispheric-rivalry model suggests why some neglect patients may be hyper-attentive to ipsilesional objects (e.g., Kinsbourne, 1977; Ladavas, Petronio, & Umilta, 1990; Smania et al., 1998). Although not always found, such ipsilesional biases may arise at the expense of selectivity for task-relevant features (Snow & Mattingley, 2006). The hemispheric rivalry account also sheds potential light on why a second lesion, opposite to the first, can sometimes lead to recovery from neglect (Brighina et al., 2003; Payne, Lomber, Rushmore, & Pascual-Leone, 2003; Vuilleumier, Hester, Assal, & Regli, 1996), presumably by partially restoring hemispheric balance (see also Koch et al., 2008).

In addition, a number of studies in healthy subjects have also provided apparent support for the ‘seesaw’ hemispheric-rivalry model. Unilateral touch, for example, can cause not only contralateral activation, but also ipsilateral deactivation in human primary somatosensory cortex, S1 (Hlushchuk & Hari, 2006; Kastrup et al., 2008). Furthermore, in the motor rather than somatosensory domain, a large body of research has demonstrated interhemispheric inhibition for motor cortex. For instance, stimulating motor cortex in one hemisphere with a pulse of transcranial magnetic stimulation (TMS) can inhibit the response of the contralateral motor cortex to TMS several milliseconds later (e.g., Daskalakis, Christensen, Fitzgerald, Roshan, & Chen, 2002; Ferbert et al., 1992). Furthermore, using prolonged low-frequency (e.g., 1 Hz) repetitive TMS to decrease excitability in one motor cortex can lead to *enhanced* excitability in the contralateral motor cortex (Plewnia, Bartels, & Gerloff, 2003; Schambra, Sawaki, & Cohen, 2003). This could be a natural consequence of release from ‘inhibition’ from the targeted hemisphere in the contralateral hemisphere, according to the rivalry account.

Returning to the somatosensory domain, one seminal paper sought to infer interhemispheric effects from behavior, specifically from subjects’ ability to detect tactile stimulation after transcranial magnetic stimulation. Seyal, Ro, and Rafal (1995) applied single pulses of TMS to the right parietal or frontal lobe, 50 ms before a perithreshold stimulus to the ipsilateral right thumb. These tactile stimuli initially project to the left somatosensory cortex, in the hemisphere opposite TMS. The authors found that subjects perceived more right-thumb stimuli (ipsilateral to the right parietal TMS) and had a lower sensory threshold for these, compared to trials with frontal TMS or no TMS. Seyal et al. (1995) interpreted their findings in the context of interhemispheric rivalry. They argued that the TMS pulses disrupted the targeted right parietal site, thus putatively ‘disinhibiting’ the left hemisphere to improve performance on the right hand.

The seesaw-rivalry theory is not the only possible account for these results, however. Several lines of evidence suggest that interhemispheric interactions may take other forms. First, the physiology of the corpus callosum does not immediately lend itself to a strict rivalry model, which assumes primarily *inhibitory* interactions between the hemispheres. Axons in the corpus callosum project almost entirely between pyramidal neurons, forming excitatory synapses (Jacobson & Trojanowski, 1974; Wise & Jones, 1976). Indeed, the predominance of excitatory interactions may underlie the effectiveness of surgical callosotomies in limiting the spread of epileptic activity, and potentially also explain why the corpus callosum is typically smaller in individuals with greater behavioral laterality (for review, see Bloom & Hynd, 2005).

Second, in contrast to the experiments cited above, several electrophysiological and imaging studies have demonstrated a bilateral SI response to unilateral tactile stimulation. In humans, intracranial recording (Noachtar, Luders, Dinner, & Klem, 1997), magnetoencephalography (MEG; see Kanno, Nakasato, Hatanaka, & Yoshimoto, 2003; Korvenoja et al., 1995; Schnitzler, Salmelin, Salenius, Jousmaki, & Hari, 1995) and functional magnetic resonance imaging (fMRI: see Hamalainen, Hiltunen, & Titievskaja, 2002; Hansson & Brismar, 1999; Nihashi et al., 2005; Polonara, Fabri, Manzoni, & Salvolini, 1999) have all shown that median-nerve or hand stimulation can partially activate regions within ipsilateral SI, in addition to contralateral SI. Furthermore, in monkeys, a series of single-cell recording studies by Iwamura and colleagues showed that some neurons in the caudal-most portion of SI display bilateral hand receptive fields (for review, see Iwamura, Taoka, & Iriki, 2001). These results seem hard to reconcile with purely inhibitory interhemispheric exchanges, even for somatosensation.

Finally, a number of TMS studies also apparently fail to support an account in terms of hemispheric rivalry alone. For example, some studies have shown that prolonged low-frequency repetitive TMS decreases excitability both ipsilaterally *and* contralaterally (Nowak et al., 2008; Wassermann, Wedegaertner, Ziemann, George, & Chen, 1998). Such bilateral decreases seem more consistent with the presence of some (normally) excitatory transcallosal interactions, rather than with interhemsipheric rivalry alone.

Importantly, to our knowledge, no TMS study of somatosensation has as yet reported both a contralateral decline and an ipsilateral improvement in tactile processing, as the rivalry hypothesis would predict. Cohen, Bandinelli, Sato, Kufta, and Hallett (1991), for instance, were the first to demonstrate that single-pulse TMS of SI can disrupt contralateral detection. Although they also measured behavior on the ipsilateral side, they found no effect of TMS there. Subsequent single-pulse (Harris, Miniussi, Harris, & Diamond, 2002) and repetitive TMS (Knecht, Ellger, Breitenstein, Bernd, & Henningsen, 2003; Satow et al., 2003) studies have confirmed this selective result. If the hemispheres were in strict rivalry, one would presumably expect the ipsilateral hand to demonstrate improved processing when the contralateral hand demonstrates a disruption, but as yet this pattern has not been reported (although it was not always tested for). Analogously, TMS in studies of the motor (Dafotakis, Grefkes, Wang, Fink, & Nowak, 2008; Kobayashi, Hutchinson, Theoret, Schlaug, & Pascual-Leone, 2004) or visual system (Chambers, Stokes, Janko, & Mattingley, 2006; Dambeck et al., 2006; Hilgetag, Theoret, & Pascual-Leone, 2001) often cause either an ipsilateral enhancement or a contralateral decrement, but rarely both complementary patterns together, despite this being the simplest prediction from strict hemispheric rivalry.

Taken together, the available data suggest that the hemispheres may not always be in direct competition, unlike the traditional ‘seesaw’ model. Rather, the nature of interhemispheric interactions seems likely to include potential excitatory influences also, and may depend on the exact task and brain regions involved (and probably the exact TMS protocols also). To further explore these issues, we returned to the classic paradigm of Seyal et al. (1995), the first study to have demonstrated that right parietal TMS can enhance ipsilateral tactile processing. Those authors interpreted their finding as evidence that single-pulse TMS disrupted the right parietal cortex, thereby ‘disinhibiting’ the left parietal cortex to improve *right-hand* detection. However, Seyal et al. (1995) did not measure performance on the *left hand*, and thus could not test whether contralateral performance was disrupted along with the ipsilateral benefit, as expected according to the standard seesaw rivalry model. We sought to extend Seyal et al. (1995) to test whether the effects in that design were truly due to interhemispheric rivalry, or whether other types of interhemispheric interplay might explain the results instead. Our methods related not only to their original study, but also to a more recent concurrent TMS-fMRI study (Blankenburg et al., 2008) of TMS effects on somatosensory processing, as explained further below.

We conducted two related experiments, presented sequentially below. In both experiments, short bursts of 10 Hz TMS (exactly as in the recent Blankenburg et al., 2008 study, so that we could potentially relate our present behavioral findings to their fMRI results) were applied on-line to the right parietal lobe on each trial, during electric stimulation of either the right or the left median nerve. The only difference between our two TMS experiments was the task involved. In Experiment 1, we asked participants to *detect* the presence or absence of perithreshold tactile stimuli, analogously to the paradigm used by Seyal et al. (1995). This allowed us to directly assess those authors’ rivalry interpretation. In Experiment 2, we asked participants to *discriminate* between two consecutive suprathreshold tactile stimuli, one slightly more intense than the other. This experiment was designed to compare the rivalry account to an account based on excitatory interhemispheric influences, which makes different predictions for the effect of TMS on performance. To anticipate, our findings argue against the traditional seesaw rivalry account for interhemispheric influences, leading to a very different account.

## 2 Experiment 1: Somatosensory detection

### 2.1 Aims and predictions

**Fig. 1:**
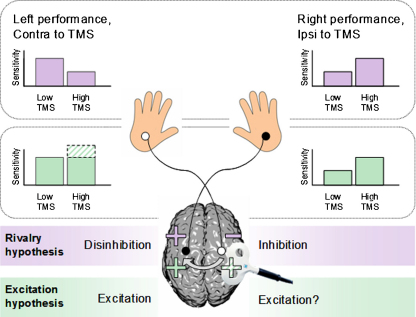
Possible predictions for Experiment 1. In Experiment 1, subjects attempted to detect weak electrical median-nerve stimuli while TMS was delivered over the right parietal lobe. In half of the trials, TMS was applied at a low intensity that should be neurally ineffective; while in the other half of the trials, TMS was applied at a high, neurally effective intensity. We had two contrasting sets of predictions for the effects of TMS in this experiment. According to the traditional rivalry hypothesis (schematically shown in purple), TMS should disrupt the targeted right parietal lobe, thereby putatively ‘disinhibiting’ the left parietal lobe, as suggested by Seyal et al. (1995). This should impair performance on the left hand but enhance it on the right. Alternatively, the more ‘excitatory’ rather than rivalrous hypothesis for interhemispheric interplay (schematically shown in green) proposes that TMS may activate excitatory interhemispheric projections (see main text), thereby increasing left parietal activity to enhance performance on the right hand. TMS might not directly modulate right somatosensory areas, but if it does, this effect might be positive also (green-shaded area), leading to enhanced performance on the left hand as well (or no change). Thus, whether or not right-parietal TMS should disrupt left-hand detection sensitivity is the primary difference between the alternative accounts, as tested here for the first time. (For interpretation of the references to color in this figure legend, the reader is referred to the web version of the article.)

In Experiment 1, we measured subjects’ tactile detection for both the right and left hands, rather than just one hand as in Seyal et al. (1995), thus allowing us to test the rivalry account more fully. We compared two sets of predictions (Fig. 1). First, if the ‘seesaw’ rivalry model applies so that when one hemisphere benefits the other faces a cost, we would expect *reduced* performance on the left hand contralateral to TMS, along with enhanced performance on the right hand ipsilateral to TMS. Seyal et al. (1995) found the latter result but did not examine the former; both are critical for a full test of the seesaw rivalry account. Second, it is possible that TMS activates excitatory interhemispheric pathways (see Section 1), so that the local effect on the directly stimulated hemisphere is transmitted to the contralateral hemisphere also. In that case, the expected pattern of enhanced right-hand performance (as found by Seyal et al., 1995) may reflect a TMS-induced signal or ‘pedestal’ being added to a weak excitatory somatosensory input to help it pass the detection threshold (as explained in more detail below). Left-hand performance (not tested by Seyal et al., 1995) might then either remain the same or improve, depending on the exact connectivity between the stimulated area of right parietal cortex and right SI, where left-hand tactile detection is likely to take place. Thus, the crucial difference between the seesaw rivalry hypothesis and alternative possibilities is that the seesaw rivalry account (as outlined by Seyal et al., 1995) suggests that a benefit in performance ipsilateral to TMS should be accompanied by a cost for the contralateral hand, while the latter possibilities do not.

### 2.2 Methods

#### 2.2.1 Participants

Fourteen right-handed volunteers (9 male, mean age 28.4 ± 12.1 SD) were recruited from the University College London Psychology Subject Pool. Each gave written informed consent and received minor monetary compensation for participating in a 1.5-h session. They were naïve to the purpose of the study and screened to rule out neurological disorders and other contraindications for TMS. The study was approved by the joint research ethics committee of the Institute of Neurology and the National Hospital for Neurology and Neurosurgery, London, UK. A subset of the following behavioral data (concerning somatosensory performance for just the right hand) has previously been reported in brief summary as a control experiment in an fMRI paper by Blankenburg et al. (2008). The present manuscript, however, is the first to report the critical extension of Seyal et al. (1995) to test performance in the left hand also, contralateral to TMS.

#### 2.2.2 Median-nerve stimulation

A pair of disposable, surface-adhesive electrodes was positioned on each participant's wrist, with the anode 0.5 cm distal to the cathode. A constant current neurostimulator (DS7A, Digitimer, Hertfordshire, U.K.) was used to apply square-wave electric pulses, each lasting 200 μs. Subjects reported sensations radiating to the thumb, index, and middle finger, verifying stimulation of the median nerve. The stimulation intensities used (see below) did not induce visible twitching.

Before beginning the task, individual median-nerve detection thresholds were determined for each subject, so as to avoid floor or ceiling effects. An initial estimate of the threshold was acquired using the staircase method (i.e., subjects reported the presence or absence of sensation as stimulation pulses were applied at gradually increasing or decreasing intensities). This estimate was then refined in a computerized two-alternative forced choice task, in which participants were asked to report which of two successive intervals contained a 500 ms, 10 Hz burst of median-nerve stimulation (see also Blankenburg et al., 2008). The task was repeated with modified intensities until subjects performed with approximately 70% accuracy. The resulting intensity was then applied during the subsequent experimental block. To account for any perceptual learning or adaptation during the task, subjects were asked to repeat the two-alternative forced-choice task between each block. If performance changed, we modified the intensity as needed to maintain ∼70% accuracy; but crucially, stimulation intensity was always constant within each block, and thus identical for the trials with high- or low-intensity TMS that were interleaved in each block, as described below.

#### 2.2.3 Behavioral paradigm

**Fig. 2:**
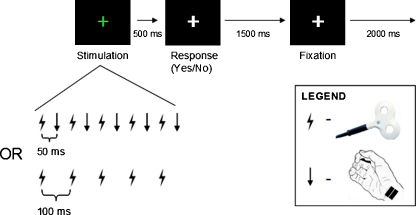
Behavioral paradigm for Experiment 1. In Experiment 1, subjects were asked to detect perithreshold tactile stimulation. Each trial began with a 500 ms burst of 10 Hz TMS over the right parietal lobe (as also used in Blankenburg et al., 2008). TMS was unpredictably applied at either 110% of motor threshold or 50% of motor threshold in a randomly intermingled trial sequence, with the low-intensity trials controlling for any nonspecific effects of TMS. On a random half of the trials, TMS pulses (shown schematically as lightning flash icons) were interleaved with median-nerve pulses (shown schematically as downward arrows), so that each pulse was separated by 50 ms (again exactly as in Blankenburg et al., 2008). On the other half of the trials, TMS was presented alone (see lower illustration). After stimulation, the fixation cross turned from green to gray and subjects had 1500 ms to respond, pressing one button if they felt median-nerve stimulation and another button if they did not. Once subjects responded, they fixated for 2000 ms before the next trial began. Two separate groups of subjects performed this experiment: one with median-nerve stimulation at the right wrist, ipsilateral to TMS (*N* = 7); and one with median-nerve stimulation at the left wrist, contralateral to TMS (*N* = 7).

To explore the effect of on-line right parietal TMS on ipsilateral or contralateral somatosensory detection for the median-nerve stimuli, we employed a psychophysical task similar to that used by Seyal et al. (1995); see Fig. 2. The task had a 2 × 2 factorial mixed design, with TMS intensity (high vs. low, see below) as a within-participant factor, and side of median-nerve stimulation (right vs. left) as a between-participant factor.

Subjects sat in a height-adjusted chair in a soundproof, darkened testing room, facing a 17-inch computer screen. They placed their chins in a chinrest and were asked to maintain fixation on a gray cross at the centre of the black screen.

At the outset of each trial, the fixation cross turned from gray to green, and a 500 ms, 10 Hz burst of TMS was applied to right parietal cortex at either 110% or 50% of resting motor threshold (as determined separately by M1 TMS; see below). Our use of online short bursts of TMS (5 pulses on each trial at 10 Hz) was equivalent to the recent concurrent TMS-fMRI study by Blankenburg et al., 2008 that we discuss later. Various 10 Hz TMS protocols have been used in past research, over a variety of different brain sites, for very different durations (e.g., for seconds to minutes, rather than in 500 ms bursts as here), and in a wide range of different stimulus, cognitive or clinical contexts (e.g., Chambers et al., 2006; De Ridder et al., 2005; Fitzgerald, Fountain, & Daskalakis, 2006; Kleinjung, Steffens, Londero, & Langguth, 2007; Lefaucheur, Drouot, Keravel, & Nguyen, 2001; Pascual-Leone et al., 1994; Pascual-Leone, Rubio, Pallardo, & Catala, 1996; Paus, Castro-Alamancos, & Petrides, 2001; Plewnia, Lotze, & Gerloff, 2003). Indeed there are so many differences between the various 10 Hz TMS protocols used in the past that it is difficult to make general statements about the exact mechanism of neural action for 10 Hz TMS, even for whether this should typically be considered ‘inhibitory’ or ‘excitatory’ in terms of local action. Accordingly we did not wish to make strong prior assumptions about the exact basis of TMS impact here. Instead we sought to exploit a particular established TMS protocol (Blankenburg et al., 2008) that has recently been shown to benefit *ipsilateral* detection (analogously to Seyal et al., 1995) for median-nerve somatosensory stimulation, in order to test for the first time our specific question of whether exactly the same TMS protocol will impair rather than enhance such detection for the *contralateral* hand, as the seesaw rivalry account should predict.

We varied the intensity of the 10 Hz TMS bursts. The 50% intensity pulses mimicked the scalp sensation and acoustic click of the high-intensity pulses without causing as much neural influence; thus, they were intended to control for any nonspecific effects of TMS (see also Blankenburg et al., 2008). The 110% intensity was chosen to be consistent with the intensity used by Seyal et al. (1995). On a random half of the trials at each intensity, the 5-pulse TMS burst was applied in the absence of median-nerve stimulation. On the other half of trials, the TMS burst was interleaved with a 500 ms, 10 Hz burst of perithreshold median-nerve stimulation, which could presumably excite the contralateral primary somatosensory cortex to which it will initially project, then secondary somatosensory cortex, and so on. On trials with median-nerve stimulation present, the first TMS pulse was followed 50 ms later by the first median-nerve stimulus, which was followed 50 ms later by the next TMS pulse, and so on (see Fig. 2). This timing aspect was also exactly as in Blankenburg et al., 2008, and importantly was held constant across the different conditions compared here.

After the burst of stimulation on each trial, the fixation cross turned back from green to gray. The subjects then had 1500 ms to respond, pressing one key to indicate that they detected tactile stimulation or another key to indicate that they did not. The mapping of these response keys was chosen by the participant at the beginning of the experimental session and remained consistent throughout. Participants were told simply to ignore the TMS as far as possible. Once the response period ended, the gray fixation cross remained on screen for 2 s until the next trial began, for a total trial duration of 4 s.

Each subject completed 4 blocks, each lasting approximately 4 min. Each block included 60 randomly intermixed trials, 15 for each of the conditions created by the 2 × 2 combination of TMS intensity (high vs. low) and median-nerve stimulation (present vs. absent) on the currently relevant hand. Seven subjects received right median-nerve stimulation, ipsilateral to TMS; while 7 different subjects received left median-nerve stimulation, contralateral to TMS. Blocks were removed from analysis if subjects performed with greater than 90% accuracy or if they detected the tactile stimulation in fewer than 10% of occasions, to avoid ceiling and floor effects, respectively. This exclusion procedure was used because tactile sensitivity fluctuated somewhat across the course of the experiment, which was only partially compensated for by adjusting wrist stimulation intensity prior to each block. Out of the 56 blocks collected in Experiment 1, only 13 blocks had to be excluded in this manner.

All stimuli were controlled using the MATLAB (Mathworks, Natick, Massachusetts, USA) toolbox Cogent 2000 (http://www.vislab.ucl.ac.uk/Cogent/) running on a conventional PC. Button-press responses were recorded online by the program used to deliver TMS and tactile stimulation.

#### 2.2.4 TMS protocol

We used a 70-mm figure-of-eight coil connected to a Magstim Super Rapid stimulator (Magstim, Dyfed, U.K.). The coil was placed tangentially to the scalp with the handle pointing posterolaterally at a 45-degree angle to the sagittal plane. Pulses induced a biphasic current with an initial anteroposterior direction. Stimulation parameters abided by current safety guidelines (Rossi et al., 2009; Wassermann, 1998).

To position the TMS coil over the right parietal cortex, we used the same approach as Seyal et al. (1995), thus allowing comparability with that study. First, we found the optimal position for stimulating the left thenar muscles, over right M1. We then defined the motor threshold as the percent of maximum stimulator output (MSO) required to produce a visible twitch in the resting left first dorsal interosseous muscle in 5 out of 10 TMS pulses. Once we located the motor hotspot and determined the threshold in this way, we moved the TMS coil in a posterior direction in steps of 10 mm, applying single pulses at 110% of the motor threshold until no twitches were detected and the participant ceased to report any sensation in the left hand. This location, generally within 2-5 cm posterior to the motor hotspot, was marked on a tight-fitting white silicone swimming cap and used for the subsequent experiment. The coil was clamped in place with a mechanical arm (Manfrotto, Bassano del Grappa, Italy), while a chin-rest kept the participant's head fixed. Two participants, one in each hand group, did not readily tolerate the high-intensity TMS (all participants were asked if this was ‘comfortable’), so for them the intensity was reduced to 5% above the motor threshold.

#### 2.2.5 Data analysis

Signal detection theory was applied to analyze the effect of TMS intensity on performance in the somatosensory task. Standard formulae were used to calculate orthogonal indices for *d*-prime (*d*′) and criterion (*c*), independently for high- and low-intensity TMS trials within each block (Macmillan & Creelman, 2005). *d*′ was defined as the difference between the *z*-transform of the hit rate and the *z*-transform of the false alarm rate, so that higher values of *d*′ reflect greater sensitivity:

$$d^{\prime }=z(H)-z(F)$$

$$c=-\frac{1}{2}[z(H)+z(F)]$$

Conversely, *c*, the bias towards a particular response, was defined as the negative mean of these measures, with a *c* of 0 reflecting an unbiased participant:

The hit rate was calculated as the probability of reporting median-nerve stimulation when such stimulation was present, while the false alarm rate was the probability of reporting median-nerve stimulation when such stimulation was absent. Following conventional procedures, hit or false alarm rates of 0 or 1 were corrected to 1/*N* or 1 − 1/*N*, respectively, where *N* was the number of trials in that block (Macmillan & Creelman, 2005).

The influence of TMS on *d*′ and *c* was analyzed using a mixed analysis of variance (ANOVA) with participants as a random effect, TMS level (high vs. low) as a within-participant factor, and side of median-nerve stimulation (right vs. left hand) as a between-participant factor. Statistical significance was set to *p* = 0.05, and values are reported as means ± standard error of the mean. All analyses were completed in Matlab 7.1 (Mathworks, Natick, Massachusetts) or SPSS 16.0 (SPSS Inc., Chicago, Illinois).

### 2.3 Results and discussion

Fourteen participants reported the presence or absence of perithreshold median-nerve stimulation (mean ± SD stimulation intensity: 2.47 ± 0.57 mA), delivered either ipsilaterally (*N* = 7) or contralaterally (*N* = 7) to on-line short bursts of right parietal TMS. The low- and high-intensity TMS parameters (selected as 50% and 110% of motor threshold) were 33.0 ± 6.2 and 71.9 ± 13.5 percent MSO, respectively. The coil was placed 3.1 ± 1.3 cm posterior to the motor hotspot.

**Fig. 3:**
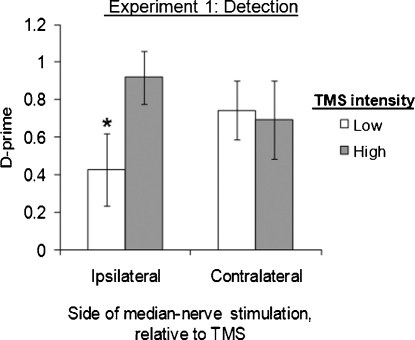
TMS enhanced ipsilateral detection. Subjects attempted to detect perithreshold median-nerve stimulation delivered either ipsilaterally (*N* = 7, right-hand group) or contralaterally (*N* = 7, left-hand group) to right parietal TMS bursts. When analyzing sensitivity (*d*′) for all factorial conditions within a 2 × 2 mixed ANOVA, we found an interaction between TMS intensity and side of median-nerve stimulation [*F*(1,70) = 5.11, *p* = 0.027], such that high- vs. low-intensity TMS affected detection more on the ipsilateral side (see leftmost two bars in plot) than on the contralateral side (see rightmost two bars). Compared to low-intensity TMS, high-intensity TMS *improved* ipsilateral detection [*F*(1,28) = 14.22, *p* = 0.001, see * in plot]; but had no effect on contralateral detection [*F*(1,42) = 0.07, *p* > 0.7]. In all data figures, error bars reflect the standard error of the mean.

There were no overall main effects of TMS intensity [*F*(1,70) = 3.45, *p* > 0.05] nor of side of median-nerve stimulation [*F*(1,12) = 0.065, *p* = 0.80] on subjects’ sensitivity, *d*′. But there was a significant interaction between TMS intensity and side, such that high- vs. low-intensity TMS affected sensitivity more on the ipsilateral side than on the contralateral side [*F*(1,70) = 5.11, *p* = 0.027; Fig. 3]. There was no such interaction when criterion was the dependent variable [*F*(1,70) = 3.73, *p* > 0.05], nor any main effects, indicating that TMS did not induce different response biases in the different conditions.

To determine the source of the significant two-way interaction for sensitivity, we separated the *d*′ data into ipsilateral and contralateral groups. Seven subjects received right median-nerve stimulation, ipsilateral to parietal TMS, and thus analogous to the classic Seyal et al. (1995) study. In this situation, high-intensity TMS significantly *enhanced* sensitivity to *ipsilateral* median-nerve stimulation, compared to low-intensity TMS over the same location [simple effect of TMS intensity: *F*(1,28) = 14.22, *p* = 0.001]. This accords with Seyal et al.’s (1995) finding of improved detection ipsilateral to right parietal TMS. Right-hand sensitivity was 0.92 ± 0.14 in high-intensity TMS trials, but only 0.43 ± 0.19 in low-intensity TMS trials (see leftmost two bars in Fig. 3). There was no such effect of TMS intensity on response bias [*F*(1,28) = 1.98, *p* > 0.1] in the ipsilateral group.

Seven different subjects received left median-nerve stimulation, contralateral to the right parietal TMS. In this situation, high-intensity TMS did *not* affect sensitivity to median-nerve stimulation, compared to low-intensity TMS over the same location [high-intensity TMS: *d*′ = 0.70 ± 0.21; low-intensity TMS: *d*′ = 0.74 ± 0.16; *F*(1,42) = 0.07, *p* > 0.7, n.s.; see rightmost two bars in Fig. 3]. This absence of any impairment in sensitivity due to contralateral TMS appears to argue against the hemispheric rivalry seesaw model, which predicts that contralateral detection should be impaired in this situation, as the inevitable corollary of ipsilateral detection being enhanced. The absence of a contralateral effect in the presence of an ipsilateral benefit led to the interaction between side and TMS intensity found in the omnibus ANOVA on sensitivity above.

TMS intensity did affect response bias for the left-hand participants, such that they tended to respond ‘present’ more often for high-TMS trials than for low-TMS trials (*c* = −0.42 ± 0.13 or 0.68 ± 0.12, respectively), leading to an effect of TMS intensity [*F*(1,42) = 42.2, *p* < 0.001].

In sum, Experiment 1 replicated Seyal et al.’s (1995) finding of significant benefits in somatosensory detection ipsilateral to right parietal TMS (here for our short bursts of on-line TMS at 10 Hz; see also Blankenburg et al., 2008). But the critical result was that we did not find any impact on contralateral detection sensitivity, contrary to the expectations of a ‘seesaw’ hemispheric rivalry account, on which ipsilateral benefits should always be associated with contralateral costs. A reviewer noted that the presence of a contralateral median-nerve stimulus may presumably change excitability in the corresponding S1; but we note that the same should also be true for the S1 to which the ipsilateral stimulus projects when that is present, so the two sides should be comparable in that particular respect.

## 3 Experiment 2: Somatosensory discrimination

In Experiment 1 we found enhanced somatosensory detection ipsilateral to right parietal TMS (thus analogous to Seyal et al., 1995, while using the exact same TMS parameters of Blankenburg et al., 2008); but we did *not* find decreased performance in the contralateral hand, contrary to what should be predicted by the rivalry seesaw. This combination of results in Experiment 1 appears more consistent with the possibility that TMS activates neurons in the TMS-targeted hemisphere that go on to exert beneficial influences on the opposite hemisphere, instead of disrupting the TMS-targeted hemisphere (which should have led to a contralateral impairment that we did not find) and thereby putatively ‘disinhibiting’ the other hemisphere. In other words, excitatory inter-hemispheric influences might potentially allow performance to improve for the hand ipsilateral to TMS without necessarily incurring a deficit for the hand contralateral to TMS, consistent with the lack of such a deficit in our findings from Experiment 1.

To test this conjecture in more detail, and compare it further with the rivalry hypothesis, we designed a second related TMS experiment but now using a different task. Instead of *detecting* perithreshold median-nerve stimuli as before, subjects were now asked to *discriminate* between the intensity of two consecutive *supra*threshold stimuli on the same hand, judging which was stronger. Predictions for this new experimental situation differed for the two hypothetical accounts of interhemispheric interplay discussed above. If, as in the traditional seesaw-rivalry theory, TMS disrupts perceptual processing in one hemisphere and enhances it in the other, we would expect that discrimination performance should again improve for the hand ipsilateral to TMS and so should presumably worsen for the hand contralateral to TMS. But if instead TMS adds a beneficial signal (or ‘pedestal’, analogous to some increase in stimulus intensity) for processing in the contralateral hemisphere, we can predict that performance on the ipsilateral hand would either remain the same or more likely should now *deteriorate* ipsilaterally, as explained below. (Performance on the contralateral hand would likely show a similar pattern, depending on the exact connectivity between the site of stimulation and the site of tactile processing within the same hemisphere; see Discussion.)

**Fig. 4:**
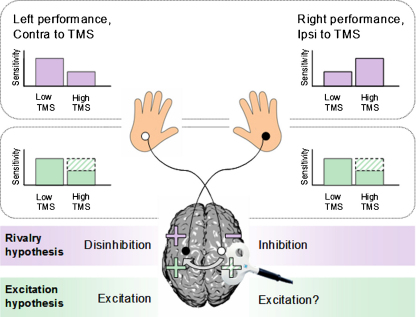
Possible predictions for Experiment 2. In Experiment 2, TMS was applied to the right parietal lobe exactly as in Experiment 1, except that subjects now discriminated between two successive electrical median-nerve stimuli of different supra-threshold intensities. TMS was applied at a low intensity in half of the trials, and at a high intensity in the other half, in a randomly intermingled sequence. As for Experiment 1, we had two contrasting sets of predictions for the effect of TMS. The rivalry model (schematically shown in purple) assumes a mutually inhibitory relationship between the hemispheres, such that a disruption in left-hand performance should correspond with an enhancement of right-hand performance, and vice-versa. The more excitatory alternative for interhemispheric interplay (shown in green) instead suggests that TMS might add a pedestal-like boost (putatively excitatory) to the contralateral hemisphere. This could lead to no change in right-hand performance (see shaded green sections in schematic plots), if the brain simply computes the absolute difference between the two electrical stimuli. But it is also possible (indeed highly plausible, based on the existing psychophysical literature, plus the control Experiment 3 here) that the brain accumulates evidence such that successful discrimination depends on the *relative* difference between the stimuli, in a Weber-like manner. In this case, adding a TMS-induced pedestal (putatively excitatory) would effectively decrease the Weber fraction, leading to *impaired* discrimination. Any effect on left-hand performance would depend on the exact connectivity between the stimulation site and somatosensory areas, but the excitatory prediction is for a trend in the same direction for both sides of the body. Thus, the major difference between the models is for the effect of TMS on right-hand performance, with the standard rivalry account predicting a benefit as in Experiment 1, but the excitatory account now suggesting a right-hand cost instead, due to a pedestal-like interhemispheric effect of TMS. (For interpretation of the references to color in this figure legend, the reader is referred to the web version of the article.)

Fig. 4 illustrates the key new predictions. In accord with previous literature (for review, see Gold & Shadlen, 2007), we assumed that the perceptual decisions required in our experiments, as in so many others, rely on evidence accumulation in favor of one alternative over the other (i.e., that the stimulus is present versus absent in Experiment 1; or that the stimulus is more or less intense in Experiment 2). For the case of Experiment 1, adding a putative TMS-induced signal (equivalent to a pedestal-like increment, so putatively excitatory) to ongoing processing in the opposite hemisphere could make it more likely for a weak, near-threshold excitatory somatosensory stimulus to exceed the threshold for responding ‘present,’ thus improving detection performance for the ipsilateral hand. But importantly, for the new intensity-discrimination task of Experiment 2, adding such a TMS-induced ‘pedestal’ to the two suprathreshold somatosensory signals that must be compared on each trial would not change the absolute difference between those two tactile stimuli. It would, however, reduce the *relative* difference. If subjects rely on the relative difference in intensity when making the intensity discrimination [as is implied by many Weber-like phenomena throughout the perceptual literature (see Gescheider, 1997), including for somatosensation, (e.g., Francisco, Tannan, Zhang, Holden, & Tommerdahl, 2008)], then the proposed pedestal-like effect of TMS should now *impair* intensity-discrimination performance, quite unlike the *enhancement* of detection that was observed ipsilaterally in Experiment 1. Thus, for Experiment 2, the crucial difference between the seesaw rivalry account and the putatively excitatory, pedestal-like possibility is that the former predicts a similar pattern to the detection task, while the pedestal account predicts that somatosensory intensity discrimination might now be *impaired* rather than enhanced by exactly the same TMS protocol.

### 3.1 Materials and methods

#### 3.1.1 Subjects

Fourteen new right-handed volunteers (6 male, mean age 26.5 ± 6.3 SD) were recruited from the University College London Psychology Subject Pool. The consent and screening process was as for Experiment 1.

#### 3.1.2 Median-nerve stimulation

Electrodes were positioned in the same manner as in Experiment 1, but since subjects were now asked to discriminate between two successive bursts of suprathreshold median-nerve stimulation, we attached two constant current neurostimulators, each generating a different intensity, to the same pair of electrodes. The staircase method was again used to provide an estimate of each subject's detection threshold. This intensity was then doubled to provide a starting value for a computerized two-alternative forced-choice task, which was designed to obtain an accurate measure of the discrimination threshold (i.e., the intensity difference between two suprathreshold stimuli that subjects could just detect). In this task, subjects were asked to report which of two successive intervals on each trial contained the more intense burst of median-nerve stimulation. We repeated this task, modifying both stimulation intensities, until subjects performed with approximately 70% accuracy. These intensities were then applied during the experimental blocks. As in Experiment 1, we sought to adjust for perceptual learning and adaptation during the task by asking subjects to repeat the two-alternative forced choice task without any TMS between each block. If performance changed, we modified the intensities as needed to maintain ∼70% accuracy. Note that we ensured that the two median-nerve intensities used for each block were constant across all TMS conditions within each block, for each participant.

#### 3.1.3 Behavioral paradigm

**Fig. 5:**
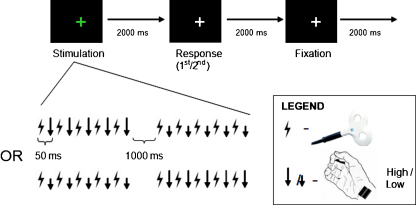
Behavioral paradigm for Experiment 2. In Experiment 2, subjects discriminated between two successive median-nerve stimuli on the same hand, differing only in intensity. Each trial comprised a 2-s stimulation period, a 2-s response period, and a 2-s fixation period before the next trial began. During the stimulation period, two consecutive bursts of TMS were applied to the right parietal lobe, both either at 110% or 50% of motor threshold. Interleaved with the TMS (lightning flash icons) were two successive bursts of suprathreshold median-nerve stimulation (arrows). On a random half of the trials, the first burst of median-nerve stimulation was stronger than the second burst. On the other half of the trials, the first burst was weaker than the second burst. After stimulation, subjects pressed one key if they felt the stronger stimulus first, and another key if they felt the stronger stimulus second. This experiment was repeated in two groups of subjects: one with right median-nerve stimulation (*N* = 7), and one with left (*N* = 7).

The tactile discrimination task (see Fig. 5) was modified from the detection task used in Experiment 1. The task had a similar mixed factorial design as before, with TMS intensity (high vs. low) as a randomly intermingled within-subject factor, plus side of median-nerve stimulation (right vs. left) as a between-subject factor. Unlike Experiment 1, we now applied two consecutive bursts of median-nerve stimulation on every trial, asking the subjects to determine which had the higher intensity (the first or the second on a given trial, which was equiprobable).

As in Experiment 1, subjects fixated on a cross at the center of a computer screen. At the start of each trial, the fixation cross turned from gray to green, and a 500 ms, 10 Hz burst of TMS (exactly as in the previous experiment, and as in Blankenburg et al., 2008) was applied at either 110% or 50% of motor threshold over the right parietal site, determined exactly as before. One second later, another 500 ms, 10 Hz burst of TMS was applied at the same intensity as the previous one, in a continuation of the same trial. Both of these bursts of TMS were interleaved with 10 Hz suprathreshold median-nerve stimuli, so that adjacent TMS and electric pulses were always separated by 50 ms (as in Experiment 1, and also as in Blankenburg et al., 2008). Thus we again used an ‘online’ TMS protocol, as in Experiment 1. On a random half of the trials, the higher-intensity median-nerve stimulus burst was presented first, while on the other half of the trials, the higher-intensity stimuli were presented second.

After the second combined burst of TMS and median-nerve stimulation, the fixation cross turned gray and participants had 2 s to respond, pressing one of two designated keys to indicate whether higher tactile stimulation was given during the first or second interval. As in Experiment 1, they were told to ignore the TMS as far as possible. After the participants responded, the gray fixation cross remained on screen for 2 s until the next trial began, for a total trial duration of 6 s.

Each subject completed 3 blocks, each lasting approximately 6 min. Each block included 60 randomly intermixed trials, 15 for each of the subconditions produced by crossing TMS intensity with whether the higher intensity median-nerve stimulation came first or second on a given trial. Each participant now completed 3 blocks instead of 4 (cf. Experiment 1) so that the total number of TMS pulses per session was within published guidelines (note that there were now two TMS bursts per trial, to correspond with the two median-nerve stimulations). Seven subjects received right median-nerve stimulation, ipsilateral to TMS, while 7 different subjects received left median-nerve stimulation, contralateral to TMS. The TMS protocol was identical to Experiment 1 and analogous to Blankenburg et al. (2008) once again.

#### 3.1.4 Data analysis

Signal detection theory was again used to analyze the results. For Experiment 2, the equation for *c* remained the same, but *d*′ was modified in the standard way (see Macmillan & Creelman, 2005) for cases with two stimulus signals, as here for the successive discrimination:

$$d^{\prime }=\frac{1}{\sqrt{2}}[z(H)-z(F)]$$

The definitions of hits and false alarms also diverged slightly from those used in Experiment 1, again to account for the different task. The hit rate was defined as the probability that the subject reported the first stimulus as more intense when the first stimulus was indeed more intense. The false alarm rate was the probability that the subject reported the first stimulus as more intense when the second stimulus was in fact more intense.

As in Experiment 1, we analyzed the results through a mixed-model ANOVA with participants as a random effect, TMS level (high vs. low) as a within-subject factor, and side of median-nerve stimulation (right vs. left hand) as a between-subject factor.

### 3.2 Results and discussion

**Fig. 6:**
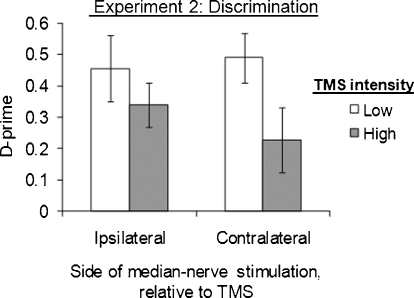
TMS disrupted suprathreshold discrimination in Experiment 2, unlike the ipsilateral benefit for near-threshold detection in Experiment 1. Subjects were now asked to discriminate intensity between two successive suprathreshold median-nerve stimuli, both delivered either ipsilaterally (*N* = 7) or contralaterally (*N* = 7) to right parietal TMS, in Experiment 2. There was a main effect of TMS intensity, such that high-intensity TMS disrupted discrimination overall [*F*(1,68) = 4.71, *p* = 0.03]. There was no interaction of TMS intensity by side of median-nerve stimulation [*F*(1,68) = 0.70, *p* > 0.4]. Thus, high intensity TMS now *impaired* performance in the intensity-discrimination task, in a manner that was statistically equivalent regardless of which median-nerve was stimulated.

14 subjects discriminated between two suprathreshold median-nerve stimuli that differed in intensity on each trial. Mean intensities for the lower and higher median-nerve stimuli were 4.95 ± 0.91 and 5.11 ± 0.98 mA, respectively, as determined for each subject prior to the experiment and between blocks. There was a significant main effect of TMS level on sensitivity, such that high- vs. low-intensity TMS now *impaired* performance [*F*(1,68) = 4.71, *p* = 0.03, Fig. 6], quite unlike the *facilitation* of detection found ipsilaterally in Experiment 1. There was no main effect of side of median-nerve stimulation [*F*(1,12) = 0.14, *p* = 0.72]. Unlike Experiment 1, there was no interaction between TMS intensity and the side of somatosensory stimulation [*F*(1,68) = 0.70, *p* = 0.41], indicating a similar outcome for both hands here. Thus exactly the same right parietal TMS protocol as in Experiment 1 (where ipsilateral detection of median-nerve stimulation was *facilitated*) now *impaired* performance for the intensity-discrimination task, regardless of which median nerve this concerned.

With response bias as the dependent variable, there was a main effect of TMS level, such that high-intensity TMS led subjects to tend to report the second stimulus as stronger [*F*(1,68) = 4.84, *p* = 0.03]. No other main effect or interaction approached significance.

In sum, exactly the same TMS that had produced (ipsilateral) enhancement of somatosensory median-nerve detection in Experiment 1 now produced reliable *impairment* of somatosensory median-nerve intensity-discrimination instead (now regardless of the side of somatosensory stimulation, see Section 5). This change from enhancement to impairment appears consistent with our proposal that TMS might add a (putatively excitatory) ‘pedestal’ increment in the level of the brain response for relevant somatosensory regions (see also Blankenburg et al., 2008, and Section 5). Such a pedestal-like increment could in principle be beneficial for a near-threshold detection task (as in Experiment 1), yet detrimental for a suprathreshold intensity-discrimination task, because the putative TMS-induced pedestal increment would effectively reduce the relative (proportional, Weber-like) difference in intensity between the two stimuli on each trial. However an anonymous reviewer correctly pointed out that we have not yet proven that an *actual* pedestal-increment in intensity of median-nerve stimulation would be detrimental to performance for a task such as Experiment 2. We addressed this with our final control experiment.

## 4 Experiment 3: Confirmation that somatosensory intensity-discrimination is impaired by an actual pedestal increment in intensity for the two stimuli to be discriminated

### 4.1 Aims and predictions

Our tentative explanation for the decrement in performance in Experiment 2 is that a TMS-induced pedestal-like phenomenon (analogous to an effective increase in intensity for each somatosensory stimulus) would act to reduce the *relative* difference in intensity between the two median-nerve stimuli that had to be compared on each trial in Experiment 2, thus making them harder to discriminate. This would accord with a range of Weber-like phenomena throughout the psychophysical literature (e.g., Gescheider, 1997; Weber, 1834), including some somatosensory examples (e.g., Francisco et al., 2008). However it remains to be shown that an *actual* pedestal increment in median-nerve stimulation intensity would impair the task of Experiment 2, and might do so in a Weber-like manner. This was the aim of our final experiment. We predicted that adding an actual pedestal increment in intensity to each of the two stimuli on each trial, in the task of Experiment 2 but now without any TMS, should impair performance; and that larger pedestals might lead to larger impairments.

### 4.2 Methods

Seven new volunteers (six male, mean age 25.3 ± 1.8 SD) participated. As in Experiment 2, subjects performed two-interval discrimination of successive bursts of median-nerve stimulation, but in this follow-up experiment no TMS was applied. On half of the discrimination trials (baseline condition), the intensities of the two stimuli were defined in exactly the same way as in Experiment 2. Thus individual detection thresholds were doubled and two surrounding intensities were determined) at which subjects discriminated with approximately 70% accuracy. For the other half of discriminations (pedestal condition), the two intensities were increased by a fixed value. This did not change the absolute difference between the two intensities, but it reduced the *relative* difference, thus mimicking the putative pedestal-like effect of TMS that we proposed above. Since we could not know the exact effective pedestal-like increment putatively due to TMS, we varied the size of the actual median-nerve pedestal here in steps of 0.1 mA across subjects (mean ± SD: 1.41 ± 0.88 mA), thereby covering a range of approximately 30–80% of the individual detection thresholds. Each subject performed 10 blocks of 20 trials in each of the two conditions, with otherwise randomized block order.

### 4.3 Results and discussion

**Fig. 7:**
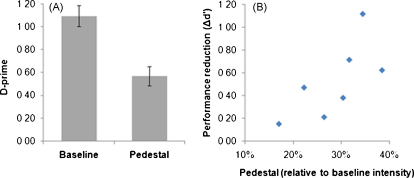
Adding an actual pedestal to both successive somatosensory median-nerve stimuli impairs discrimination in Experiment 3. In our behavioral follow-up control study (Experiment 3), seven new subjects were asked to discriminate intensity between two consecutive suprathreshold median-nerve stimuli, as in Experiment 2 but now without TMS. For half of these discriminations, the intensities of the two stimuli were set in the same way as in Experiment 2. For the other half of the discriminations, we increased both intensities by a fixed value (different fixed pedestal values for different subjects, see main text). This pedestal did not change the absolute difference between the two intensities, but it reduced their relative difference. Thus, the pedestal was intended to mimic the suggested pedestal-like effect of TMS that might impact on Weber fractions for the discrimination. Subjects performed worse in the pedestal condition (see A) than in the baseline condition (*p* = 0.02, Wilcoxon signed-rank test, two tailed). Furthermore, as shown in the subject-by-subject scatterplot in B, the decrease in performance correlated with the size of the pedestal (*R*_*Spearman*_ = 0.79, *p* = 0.048), in a Weber-like manner; see main text.

In the baseline condition, mean intensities for the lower and higher median-nerve stimuli were 4.92 ± 0.88 and 5.11 ± 0.91 mA, respectively. In the pedestal condition, a mean intensity of 1.41 ± 0.88 mA was added to each stimulus. Discrimination performance (*d*′) was significantly lower (see Fig. 7a) in the pedestal condition (mean 0.57 ± 0.22 SD) than in the baseline condition (mean 1.09 ± 0.24 SD, *p* = 0.02, Wilcoxon signed-rank test, two tailed). Furthermore, across subjects, the decrease in performance was positively correlated with the size of the individual pedestal (*R*_Spearman_ = .79, *p* = 0.048; Fig. 7b). These results confirm, as expected, that for the same median-nerve two-interval discrimination task as in Experiment 2, an actual pedestal increment in the two intensities that have to be discriminated on each trial leads to impaired performance, with this impairment tending to be larger for larger pedestals. This is in accord with the standard expectations of Weber's law, but more importantly for present purposes in also in general accord with our suggestion of a pedestal-like effect due to TMS, as discussed further below.

## 5 General discussion

We studied the effects of right parietal repetitive TMS on somatosensory processing for median-nerve stimulation at either hand. Our paradigms allowed us to compare different accounts of interhemispheric interaction. The first is the traditional view of seesaw-like hemispheric rivalry, in which each hemisphere is thought to inhibit the other, so that any decrement in somatosensory processing for one hand should be accompanied by a benefit for the other, and vice-versa. The second is an alternative perspective that includes the possibility of excitatory rather than only rivalrous interhemispheric interactions for somatosensory processing. Our findings appear more consistent with the latter model, suggesting that interpretation of the classic Seyal et al. (1995) somatosensory TMS study in terms of hemispheric rivalry alone might have been premature. Neither our somatosensory detection task (Experiment 1) nor the intensity-discrimination task (Experiment 2) exhibited a seesaw pattern, in which improvement for one hand corresponded with disruption on the other. Rather, in terms of interhemispheric interplay, the pattern of results here suggests that TMS over the right parietal lobe (at least for short bursts at 10 Hz, as used here) might convey some excitatory input to the contralateral side, as we explain below.

To our knowledge, this is the first study to use online brief TMS bursts to investigate interhemispheric effects on somatosensory processing. All previous studies used either single-pulse TMS (e.g., Harris et al., 2002; Oliveri et al., 1999) or an offline approach using prolonged repetitive TMS with comparisons made pre/post this prolonged intervention (e.g., Knecht et al., 2003; Satow et al., 2003). Single-pulse TMS inherently limits stimulation to a very narrow time window, while offline TMS is an indirect tool that might involve compensatory responses to prolonged disruption. By applying short TMS bursts to the right parietal lobe for the duration of the tactile stimuli, we hoped to modulate any interhemispheric interactions ‘online’ over the extent of their occurrence. We note also that our present short online TMS bursts (5 pulses at 10 Hz) were equivalent to those used in a recent concurrent TMS-fMRI study (Blankenburg et al., 2008), as discussed further below. Moreover, this aspect of our TMS protocol was held constant across our two TMS experiments here, so cannot by itself explain the dramatically different outcome we found for Experiments 1 versus 2.

### 5.1 Rivalrous vs. excitatory interhemispheric influences

We performed two related TMS experiments. The first was analogous to the study by Seyal et al. (1995), the sole prior demonstration that right-parietal TMS can improve ipsilateral (right-hand) tactile processing. Importantly, however, we extended that study here to examine contralateral (left-hand) processing as well as ipsilateral (right-hand) processing in different groups of subjects, while using short on-line TMS bursts as in Blankenburg et al. (2008). To explain their data, Seyal et al. (1995) had invoked the rivalry hypothesis, proposing that TMS may have disrupted the (right-parietal) cortex under the coil, thereby ‘disinhibiting’ the opposite hemisphere in the manner of a seesaw. By measuring behavioral performance for *both* hands separately for the first time here, we could test this rivalry interpretation. Despite using somewhat different TMS parameters than Seyal et al. (1995; but exactly the same parameters as Blankenburg et al., 2008), we replicated Seyal et al.’s core finding of behavioral improvement in somatosensory detection *ipsilateral* to TMS. However, we did *not* see a complementary decrement *contralateral* to TMS. This new evidence argues against a strict ‘seesaw’ rivalry interpretation. It also complements several previous findings from TMS studies on the somatosensory system (Cohen et al., 1991; Harris et al., 2002; Knecht et al., 2003; Satow et al., 2003), the motor system (Dafotakis et al., 2008; Kobayashi et al., 2004), and the visual system (Chambers et al., 2006; Dambeck et al., 2006; Hilgetag et al., 2001). Although not always noted, those studies in fact all demonstrated either ipsilateral improvements or contralateral declines in performance after TMS, but never both together. This overall pattern—like our own current findings—seems to argue against the traditional rivalry interpretation.

Our second TMS experiment was designed to further test the rivalry hypothesis, and to compare it with an account including possible excitatory interhemispheric influences. Instead of detecting perithreshold stimuli, as in Seyal et al. (1995) and our own Experiment 1, volunteers now discriminated intensity for two successive suprathreshold stimuli. If the seesaw-rivalry model applied to tactile processing in general, TMS should presumably again cause ipsilateral enhancement (and might also be expected to produce a decrement for the contralateral hand, although that had not been found in Experiment 1). Instead, we now found that the same TMS now impaired rather than facilitated performance, regardless of hand. Again, these findings do not seem to accord with a seesaw-rivalry account of hemispheric interactions such as that proposed by Seyal et al. (1995).

The results of both experiments do, however, appear to fit an account including possible excitatory interactions between the hemispheres. In this model, TMS bursts over the right parietal lobe may activate excitatory interhemispheric pathways, thereby injecting some (TMS-induced) ‘signal’ into left somatosensory cortex. Such an injection could in principle improve *detection* for the right hand, by providing a ‘pedestal’ on which a near-threshold right-hand input could stand to help it pass just above threshold. This could explain both Seyal et al.’s (1995) ipsilateral detection-benefit finding and our own for Experiment 1. For right-hand *discrimination*, however, adding a pedestal-like TMS input would be either ineffective or more likely could actually become detrimental to performance (see Fig. 4). Adding a pedestal to the response for both of two successive somatosensory stimuli should not change the absolute difference between them. It could, however, reduce the *relative* difference (akin to a Weber fraction), thereby making discrimination more difficult. In accordance with this idea that our high-intensity TMS might induce a pedestal-like effect, we found in Experiment 2 that TMS now *reduced* rather than facilitated subjects’ somatosensory performance. Thus, exactly the same TMS protocol that had improved ipsilateral detection of near-threshold median-nerve stimulation (Experiment 1) did not improve intensity-discrimination of suprathreshold stimuli (Experiment 2), but rather impaired this. These results agree with our (putatively excitatory) ‘pedestal’ account, but contradict the seesaw-rivalry account. Moreover, in a follow-up non-TMS control study (Experiment 3), we directly confirmed that an actual pedestal-increment in median-nerve intensity for the two stimuli that had to be discriminated did indeed lead to impaired performance, in a Weber-like manner, as we expected.

The effects of TMS on *contralateral* hand performance also did not support a simple hemispheric rivalry account. We found that high- vs. low-intensity TMS did not affect contralateral detection (contrary to a seesaw-like rivalry prediction), but did disrupt contralateral discrimination. Speculatively, the effect of TMS on contralateral discrimination might reflect an excitatory projection from the stimulated region of parietal cortex to the ipsilateral somatosensory areas that underlie discrimination. Intrahemispheric excitation might perhaps be more likely to affect discrimination than detection, as more regions [e.g., secondary somatosensory cortex (SII) in addition to SI] may be required for discrimination than for detection (Lin et al., 2003; see also our section below discussing mechanisms). Involvement of SII in discrimination might also explain the more bilateral pattern of results found here in Experiment 2, since each SII is known to integrate information from *both* sides of the body (Iwamura et al., 2001).

The above results all refer to *d*′, a pure measure of perceptual sensitivity (Macmillan & Creelman, 2005). For completeness, we also calculated subjects’ response bias, *c*, although these results are less important. In Experiment 1, *c* was only affected when subjects had to detect perithreshold stimuli on the left hand, contralateral to TMS. In this condition, subjects tended to respond ‘present’ more frequently in high-intensity TMS trials than in low-intensity TMS trials. Speculatively, this might reflect TMS evoking slight tactile sensations that subjects could potentially confuse with real tactile stimulation. Although we made every effort to position the TMS coil so that it did not induce scalp sensations, those cannot be completely ruled out. Similarly, in Experiment 2, high-intensity TMS sometimes led subjects to report the second stimulus as stronger. This could potentially be explained by a stronger memory for any sensations caused by the more recent TMS burst, compared to the first TMS burst. It is important to note, however, that since *d*′ and *c* are independent measures, the sensitivity results that were the focus of our investigation cannot be contaminated by any changes in response bias, so we need not be concerned any further with the latter bias effects.

### 5.2 Potential neural mechanisms

Although our dependent variables were purely behavioral, we briefly speculate on potential neural mechanisms. TMS has been employed for more than 20 years (Barker, Jalinous, & Freeston, 1985), but its physiological basis is not yet fully understood. Recently, several research groups have combined TMS with either positron emission tomography (e.g., Chouinard, Van Der Werf, Leonard, & Paus, 2003; Paus et al., 2001) or fMRI (e.g., Bestmann, Baudewig, Siebner, Rothwell, & Frahm, 2004; Bohning et al., 1998; Ruff et al., 2008, 2009; see Driver, Blankenburg, Bestmann, Vanduffel, & Ruff, 2009 for review). These studies have consistently indicated that TMS does not affect only the local stimulation site in isolation. Instead, TMS can have an impact on extended functional networks, including areas remote from but interconnected with the target site (for discussion, see Bestmann, 2008; Ruff, Driver, & Bestmann, 2009), and even in the opposite hemisphere to the TMS site (see Driver et al., 2009).

Here we proposed that right parietal TMS might modulate ipsilateral tactile processing by stimulating, rather than disinhibiting, left somatosensory cortex. This suggestion receives some direct support from a recent combined TMS/fMRI study (Blankenburg et al., 2008). That study used an identical TMS protocol to the one used here, applying 10 Hz TMS bursts over right parietal cortex at 110% or 50% of motor threshold. On half the trials, TMS was interleaved with suprathreshold median-nerve stimulation to the right wrist; on the other half of the trials, TMS was applied alone. Unlike here, participants did not perform any behavioral task during Blankenburg et al.’s (2008) scanning. But importantly, the fMRI data acquired concurrent with TMS revealed that right parietal TMS bursts *enhanced* left SI BOLD signal during right-wrist somatosensory input. In other words, exactly the same right-parietal TMS protocol as used here enhanced the differential response of left SI to the presence versus absence of right median-nerve stimulation, analogous to the detection benefit observed here (Experiment 1) and in Seyal et al. (1995).

We should note that both the current study and that of Blankenburg et al. (2008) used high-frequency (10 Hz) online TMS in short bursts, in the context of median-nerve stimulation. It is possible (indeed likely) that the interhemispheric excitation we inferred might be specific to such parameters. It could be interesting to test how these results might differ from either single-pulse TMS [as used by Seyal et al. (1995)] or a lower-frequency, more prolonged repetitive TMS protocol. At least when applied offline in the context of pre/post treatment comparisons, prolonged low-frequency repetitive TMS (e.g., at 1 Hz) is thought to reduce rather than increase excitability of the targeted cortex, unlike the short bursts of 10 Hz TMS applied here (Fitzgerald et al., 2006). As mentioned in our Introduction, repetitive TMS at 10 Hz has been used in a variety of different paradigms, for different durations and intensities, over different brain sites, and in different cognitive or clinical contexts (e.g., Chambers et al., 2006; De Ridder et al., 2005; Fitzgerald et al., 2006; Kleinjung et al., 2007; Lefaucheur et al., 2001; Pascual-Leone, Valls-Sole, Wassermann, & Hallett, 1994; Pascual-Leone et al., 1996; Paus et al., 2001; Plewnia, Bartels et al., 2003; Plewnia, Lotze et al., 2003). But importantly we kept our on-line TMS protocol, with short bursts at 10 Hz, exactly the same between Experiments 1 and 2, so there were no differences in the TMS protocol that might otherwise explain the dramatic difference in outcome.

We did not use an offline TMS protocol at a lower frequency, as it would seem difficult to directly compare an online protocol at one frequency (e.g., bursts of 10 Hz TMS on each trial) to an offline protocol at a different frequency (e.g., prolonged 1 Hz TMS). Nevertheless, it remains possible that a prolonged off-line ‘inhibitory’ TMS protocol might have revealed some interhemispheric rivalry. But even if that were the case, our primary conclusion would remain the same: namely that the traditional seesaw rivalry model cannot account for *all* interhemispheric interactions during somatosensory processing.

Whether through cortical or subcortical connections, it appears that right parietal TMS can enhance activity interhemispherically in left SI (as shown directly with concurrent TMS-fMRI by Blankenburg et al., 2008, consistent with the pedestal-like behavioral effect on ipsilateral somatosensory detection found here in our Experiment 1). The effects within the TMS-targeted right hemisphere, however, remain less clear. Blankenburg et al. (2008) found no reliable activity changes under the right-parietal TMS coil, whether or not they stimulated the median nerve concurrently with TMS. They cautioned, however, that a reduced magnetic resonance signal-to-noise ratio near the TMS coil might explain this apparent null effect. Extrapolating (speculatively) from our current behavioral data, one possibility is that right parietal TMS might have increased excitability in right SII, without affecting SI. In an MEG study, Lin et al. (2003) charted the response of somatosensory areas to contralateral electrical stimuli of varying intensity. They found that perithreshold electrical stimuli (like those used in Experiment 1's detection task here) affected SI alone, while stimuli at twice the sensory threshold (like those used in Experiment 2's discrimination task here) elicited maximal responses from SII, in addition to SI. Thus, SII might be involved in discrimination of suprathreshold stimuli (as in Experiment 2 here) more than for detection of near-threshold stimuli (as in Experiment 1 here). If our TMS paradigm influenced right SII to a greater extent than right SI, we might therefore expect to find a contralateral behavioral effect only in the discrimination task, as we in fact observed when comparing Experiments 1 and 2 for the left-hand contralateral to the right parietal TMS.

For the future, an interesting extension of the current experiment might be to include trials in which both median nerves are stimulated concurrently, instead of the unilateral stimulation presented here. By provoking competition between concurrent stimuli on opposite sides of space, this type of study might potentially provoke more hemispheric rivalry. Pascual-Leone et al. (1994), Pascual-Leone, Valls-Sole et al. (1994) and Hilgetag et al. (2001) found that parietal repetitive TMS impaired detection of a contralateral *visual* stimulus only when another stimulus was presented simultaneously on the ipsilateral side. Those findings were interpreted as evidence for interhemispheric rivalry in the context of competing bilateral stimulation, analogous to many clinical studies of the ‘unilateral extinction’ phenomenon during double simultaneous stimulation (see Driver, Vuilleumier, & Husain, 2004). The Pascual-Leone et al. (1994), Pascual-Leone, Valls-Sole et al. (1994) and Hilgetag et al. (2001) studies were only performed in the visual domain, however, and to our knowledge no closely similar work has yet been performed for somatosensory processing. It remains conceivable that in a TMS paradigm like the current Experiment 1, right parietal TMS would lead to extinction of contralateral somatosensation only when ipsilateral stimuli are presented concurrently, even though it did not harm detection of contralateral stimuli presented alone, as studied here. If so, the ‘seesaw’ model might still potentially apply, but only to situations with bilateral (i.e., competing) tactile stimuli. But once again our primary conclusion that seesaw-like hemispheric rivalry cannot explain *all* interhemispheric interactions would still hold. It also remains possible that even with concurrent bilateral somatosensory inputs, one might still find clear TMS evidence against the rivalry hypothesis. Applying TMS to both parietal lobes simultaneously (or to left rather than right parietal cortex alone) would provide further directions for extensions of the present work also.

## 6 Conclusion

This study examined the possible interhemispheric interactions underlying somatosensory processing of median-nerve stimulation in the context of TMS interventions. The results of our two TMS experiments argue against the notion that the hemispheres must invariably engage in a seesaw-type rivalry, suggesting instead that excitatory interactions might also play a role. There are probably many varieties of interhemispheric interaction, depending on the task at hand (Bloom & Hynd, 2005). In some cases, the hemispheres might compete, while in other cases they cooperate. Future studies could extend the TMS paradigms introduced here, which themselves built on the pioneering work of Seyal et al. (1995), to study how targeting a specific brain region in one hemisphere can affect processing in both the same and the opposite hemisphere.
